# Complete genome sequence of the *Clostridium difficile* LCL126

**DOI:** 10.1080/21655979.2021.1894798

**Published:** 2021-04-25

**Authors:** Jianfeng Wang, Chu Yang, Chao Zhang, Xiaoyan Mao, An Lizhe

**Affiliations:** a School of Life Sciences, Lanzhou University, Lanzhou, Gansu Province, China; b Laboratory of Clostridium, Lanzhou Institute of Biological Products, Lanzhou, Gansu Province, China

**Keywords:** *Clostridium difficile* LCL126, genome, virulence analysis, methylation, function annotation

## Abstract

*Clostridium difficile* (*C. difficile*) is a kind of obligate anaerobic gram-positive *Bacillus* related with intestinal diseases and antibiotic treatment. In present study, the *C. difficile* genome was studied employing met genomic technology. Genome sequencing identified *C. difficile* LCL126 has total size of 4,301,949 bp with a 27.97% of GC content. Specifically, 4119 predicted coding genes, 188 repeat sequences, 13 prophages and 8 gene islands were detected. Additionally, gene function analysis aspect of the function annotation, effector, and virulence were concluded that total of 3367 cluster of orthologous groups of proteins genes and classified into 24 categories, while the most outstanding class was metabolic process (1533) and catalytic activity (1498). The carbohydrate-active enzymes have been detected 127 genes, pathogenicity analysis revealed that 133 reduced and 22 increased virulence (hypervirulence) genes, while 54 unaffected and 10 loss of pathogenicity genes were found. Furthermore, perform the visualization and methylation expression were revealed that the dominant types comprised m4C, m5C, and m6C with the number of 6989, 36,666, and 3534, respectively. Overall, whole genome sequence information of *C. difficile* LCL126 was obtained and functional prediction was revealed its possible toxicological potential genes existence.

## GRAPHICAL ABSTRACT

**Figure:**
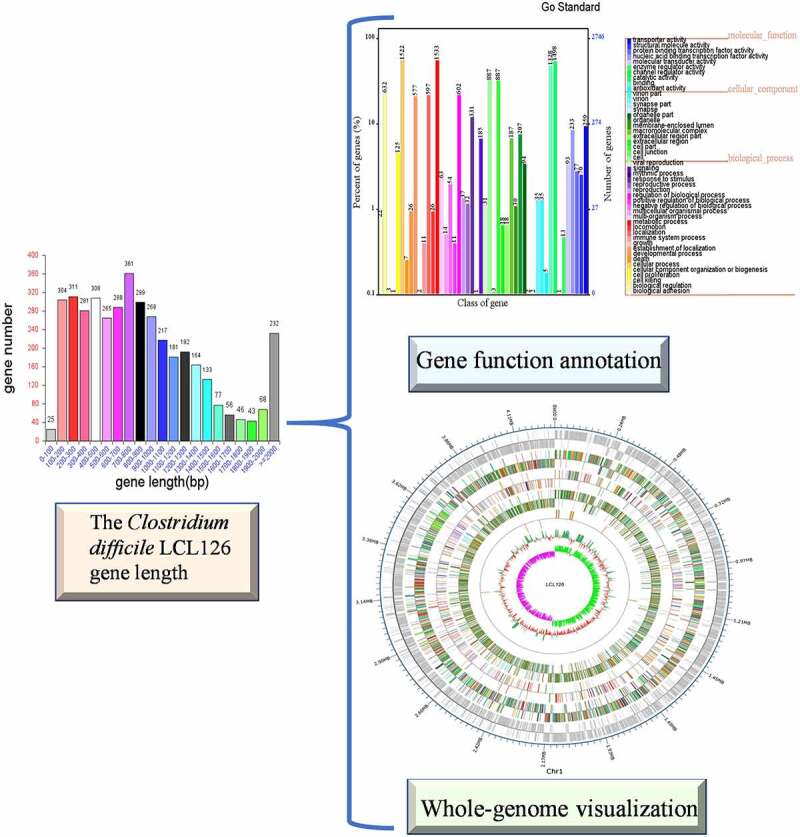

## 1. Introduction

*Clostridium difficile* (*C. difficile*) is a kind of obligate anaerobic gram-positive *Bacillus*, and considered as main causative agents of intestinal diseases related with antibiotic treatment, which can cause series of diseases such as clinical manifestations with acquired diarrhea and pseudomembranous enteritis [1,2]. The incidence of *C. difficile* infection within hospital and communities have risen prominently during the past 20 years, mainly derived from the virulence of toxins [3,4]. Alcala et al. reported that *C. difficile* becoming the quite general phenomenon of diarrhea in developed countries hospital, accounting for probably 25% of antibiotic-associated diarrhea [5], and the U.S. Disease Control and Prevention Centers has been designated the strain *C. difficile* act as an urgent threat to human health [6].

Interestingly, the feature worthy noting that self of *Clostridium* is not a pathogen while *Clostridium* infection is formed attributed by producing various toxins [7]. The various pathologies noticed from *Clostridium* infections are usually related to the toxin produced types. Clinically isolated from *Clostridium* has been classified as five toxin types from A to E in line with the major secreted lethal toxins. Each toxin can cause specific disease in human and animals from mild poisoning to potentially severe life-threatening, and the severity of symptoms varied might be partly attributable to the toxin production level [6,7]. Especially, the pathogenesis of *C. difficile* is reported in view of the action of major toxins by toxic A of *C. difficile* (*TcdA*) and toxic B of *C. difficile* (*TcdB*) encoded which disturb the intestinal epithelium powerful monoglycosy-transferase [3]. *TcdA* is an enterotoxin that can cause intestinal mucosal tissue damage and led to hemorrhagic fluid secretion, although *TcdB* lacks obvious enteric toxicity, and it is a powerful cytotoxin, most of entero-toxigenic strains producing TcdA and TcdB (A^+^ B^+^), and TcdA express missing A^−^ B^+^ strains can also cause clinical disease [8]. The typical toxin producer strains of *tcdA* and *tcdB* are found in a 19.6 kb DNA element on a similar chromosome and are called pathogenic locus [9,10], has so far been found only in pathogenic strains which inactivate the guanosine triphosphate (GTP) binding protein, caused a series of chain reactions that eventually lead to diarrhea as well as colitis from mild to life threatening [11].

The production of toxins varies greatly between distinct strains and seems to be highly affected by environmental conditions, comprising the nutrients availability and temperature changes [4]. The methods to explore the molecular perspective of *C. difficile* constantly developed, for instant pulsed-field gel electrophoresis (PFGE), multilocus variable-tandem repeat, and sequence typing, single region PCR karyotype analysis and amplified fragment length polymorphism (AFLP) [12,13]. However, these approaches application limited due to long turnaround duration, high cost while poor reproducibility and portability, as well as difficulty in data analysis. Subsequently, advanced technology developing with binary typing [14,15] and single-molecule real-time (SMRT) sequencing [16]. Recently, high-throughput sequencing genomics has been applied owing to advantages aspect of shorten turnaround duration, simplicity and distinguishability as well as portability [15].

The objective of the present study was to identify the whole genome sequencing of *C. difficile* LCL126 with attention to phylogenetic using genome analysis. In addition, to identify the information about the co-evolution and diversity of phages and bacteria, as well as attempt to elucidate the mechanism of toxicity from the perspective of genomics and metabolomics.

## 2. Materials and methods

### 2.1. Bacterial strains and DNA extraction

The *C. difficile* LCL126 strain was provided by the *Clostridium* Laboratory of Lanzhou Institute of Biological Products (Gansu, China). Genomic DNA extracted using SDS method [17]. Agarose gel electrophoresis was performed to check the purity and stability, and then further constructed database based on Pacbio and Illumina platforms. After qualified the library, carry out the selection by PacBio Sequel and Illumina NovaSeq PE150 according to the effective concentration and target data at the Beijing Novogene Bioinformatics Technology Co., Ltd., China.

### 2.2. Genome composition analysis

Genome component analysis was performed based on the valid data after quality control of each sample, and using SMRT Link v5.0.1 software to assemble the reads [17]. Align the reads to the assembled genome sequence and count the distribution were conducted to identify the sequencing depth mapping and longest sequence. Finally, by comparing the original data to the preliminary assembly sequence and optimize the assembly results by arrow software. Subsequently, perform coding and repeated sequence prediction on the newly sequenced genome by using software Gene-MarkS (Version 4.17) [18] and Repeat-Masker (Version open-4.0.5) [19]. Based on the sequence composition, IslandPath-DIOMB (Version 0.2) [20] and phiSpy (Version 2.3) [19] were used to predict gene islands and prophages that were related to a variety of biological functions including pathogenicity and environmental adaptability.

### 2.3. Genome function analysis and visualization display

Diamond comparison of the protein sequence of the predicted gene with each functional database and the result filtering and annotation. Functional analysis is mainly carried out from the following: gene ontology (GO) annotation based on cell components, molecular functions, and biological processes [21]. Kyoto encyclopedia of genes and genomes (KEGG) [22] annotations were including data on genomes, chemical molecules, and biochemical systems, aspect of metabolic pathways (KEGG PATHWAY), drugs (KEGG DRUG), diseases (KEGG DISEASE), functional models (KEGG MODULE), gene sequences (KEGG GENES), genomes (KEGG GENOME), and etc. Annotation of cluster of orthologous groups of proteins (COG) [23] was constructed according to the systematic evolutionary relationship classification of encoded proteins of the complete genome. Carbohydrate-active enZYmes database (CAZy) [24] annotation was evaluated based on the family of related enzymes that can catalyze carbohydrate degradation, modification, and biosynthesis, mainly contains five categories of glycoside hydrolases (GHs), glycosyl transferases (GTs), polysaccharide lyases (PLs) and carbohydrate esterases (CEs), and oxidation reductase (Auxiliary Activities, AAs). In addition, to comprehensively predict whether the protein sequence was a secreted protein and the effector protein using SignalP (Version 4.1), TMHMM (Version 2.0 c) [25] and EffectiveT3 (Version 1.0.1) [26]. Furthermore, pathogen-host interactions database (PHI) annotations were performed to identify virulence or pathogenicity [27].

Finally, the SMRT Link v5.0.1 [17,18] was used to perform methylation site detection and possible methylation transferase-recognized nucleotide motifs on the final genome assembly results (motif) prediction. For the assembled genome sequence of the sequenced sample and combined with the prediction result of the coding gene were performed by the using of Circos software [28] and then display the whole genome map of the sample genome.

## 3. Results and discussion

*Clostridium difficile* has attracted more and more attention in recent years due to its high prevalence, plasticity and virulence potential. The genomic diversity of *C. difficile* has been continuously explored with the advancement of biotechnology, and there is a close relation between metabolism and virulence. Based on this background, this study focuses on the *LCL126* strain through genome sequencing and trying to reveal the possible potential toxic mechanism from genome component analysis and functional annotation.

### 3.1. The Genome overview component characteristics of Clostridium difficile LCL126

**Figure 1.**
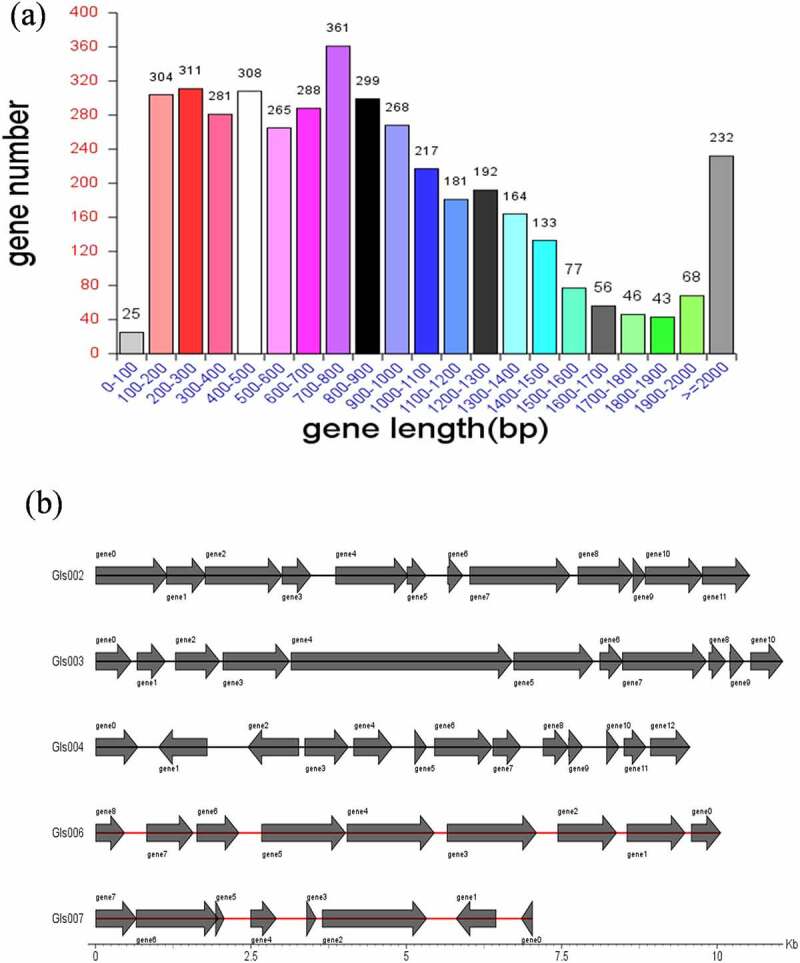
The *Clostridium difficile* LCL126 gene length statistics, the abscissa is the gene length and the ordinate is the number of the corresponding genes (a); and the statistical map of gene distribution in gene islands (b)

The identified total size of the genome *Clostridium difficile* LCL126 was 4,301,949 bp with a 27.97% of GC content using high-throughput sequencing, and including a 4,267,198 bp (28.93% GC) circular chromosome and one circular plasmids [plas1 (34,751 bp)] (Figure 1a). The number of total 4119 predicted coding genes were found with average gene length of 875 bp (account total length 83.76%). One hundred twenty-four RNAs (89 tRNAs, 11 rRNAs (5s denovo), 12 rRNAs (5s and 23s denovo) were obtained. A total 188 repeat sequence were detected comprising 105 long terminal repeat array, 31 DNA transposable, 16 short and 34 long interspersed nuclear elements as well as 2 rolling circle. The number of total 13 prophages was predicted with total length 638,171 and average 49,090.1, a total of 17 clustered regularly interspaced short palindromic repeat sequences (CRISPR) array was predicted with average length 543.412. The gene island (GI) prediction sequence was obtained number of 8 GI with 187,976 bp total and 23,497 bp average length, the statistical map of gene distribution in gene islands is presented in Figure 1b. Whole genome of *C. difficile* identified 622–3000 genes and 4.1–4.3 molecular base pairs (Mbp) length attributed to higher plasticity and degree of recombination, the difference was varied due to techniques and strains [29,30]. Some bacteriophages might be involved in regulate bacterial toxin genes, thereby altering virulence-related phenotypes while the specific role of prophages is unclear but they were involved in the virulence and pathogens evolution [31–33].

### 3.2. The genome function study of Clostridium difficile LCL126

**Figure 2.**
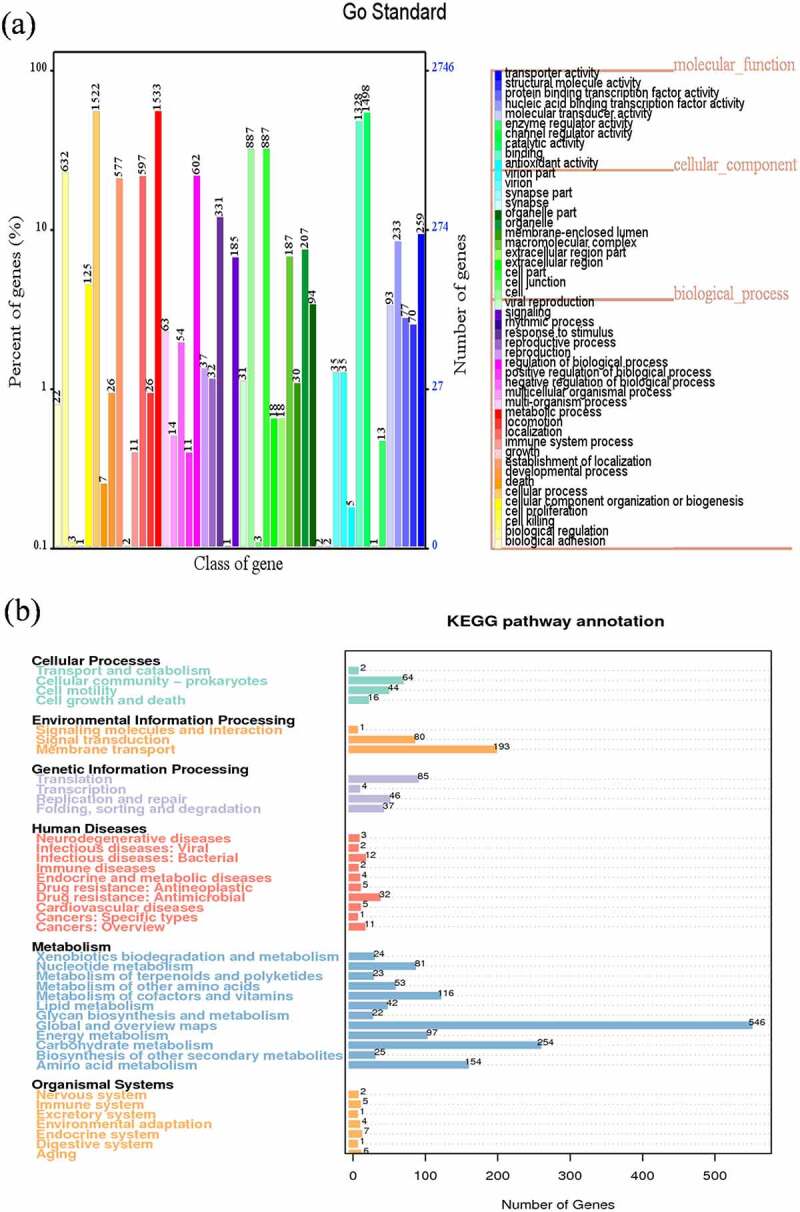
Gene function analysis of *Clostridium difficile* LCL126 based on Gene Ontology (GO) annotation: the abscissa represents the GO function classification on the sample annotation, the right ordinate represents the number of genes on the annotation, and the left ordinate represents the percentage of the number of genes on the annotation to all coding genes (a); and Kyoto Encyclopedia of Genes and Genomes metabolic pathway classification: and the number on the bar graph represents the number of genes in the annotation, and the other axis is the code of each function class of level1 in the database (b)

Gene function analysis of *Clostridium difficile* LCL126 was demonstrated in view of three aspects: function annotation, effector and virulence or pathogenicity analysis. First of all, perform functional annotations through different functional databases. In GO annotation, total number of 12,427 was detected and most outstanding class was cell (887), catalytic activity (1498) and metabolic process (1533) during classified ontology of cellular component, molecular function and biological process (Figure 2a). The KEGG annotation were shown 3175 genes and classified six mainly pathways of cellular processes, environmental information processing, genetic information processing, human diseases, metabolism, and organismal systems (Figure 2b). The predominant pathway was global and overview maps and carbohydrate metabolism with 546 and 254 unigenes, followed by membrane transport and amino acid metabolism with 193 and 154 unigenes, 116 unigenes of metabolism of cofactors and vitamins, while translation and cellular community-prokaryotes account 85 and 64 unigenes. As to COG annotation was identified total of 3367 genes and classified into 24 categories, which most abundant term was Transcription with 392 array, followed main richness term was General function prediction only (286) and Carbohydrate transport and metabolism (283) (Fig. S1a). Additionally, CAZy database obtained total 127 genes including 23 Carbohydrate-related modules (CBM), 12 CEs, 63 GHz and 29 GTs (Fig. S1b).

Meanwhile, the number of signal peptide, transmembrane structure, and secreted proteins were 84, 993, and 54. Among all the 4119 genes encode, the predicted numbers of T3SS effector and non-T3SS effector proteins were 79 and 4040, respectively. The virulence or pathogenicity analysis was revealed that 133 identified genes belong to reduced virulence, 22 increased virulence (hypervirulence) was found, while 54 unaffected pathogenicity and 10 loss of pathogenicity genes was detected (Fig. S1c).

### 3.3. Genome methylation and visual analysis of Clostridium difficile LCL126

**Figure 3.**
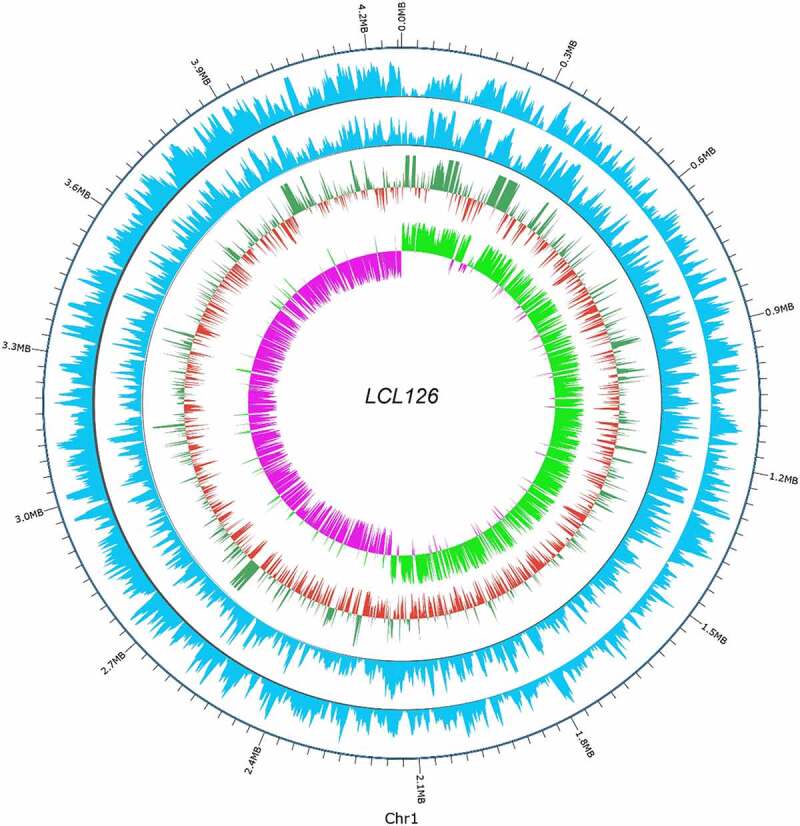
Visualization of apparent modification distribution methylation circle of *Clostridium difficile* LCL126, each circle from outside to inside represents the position of the genome

**Figure 4.**
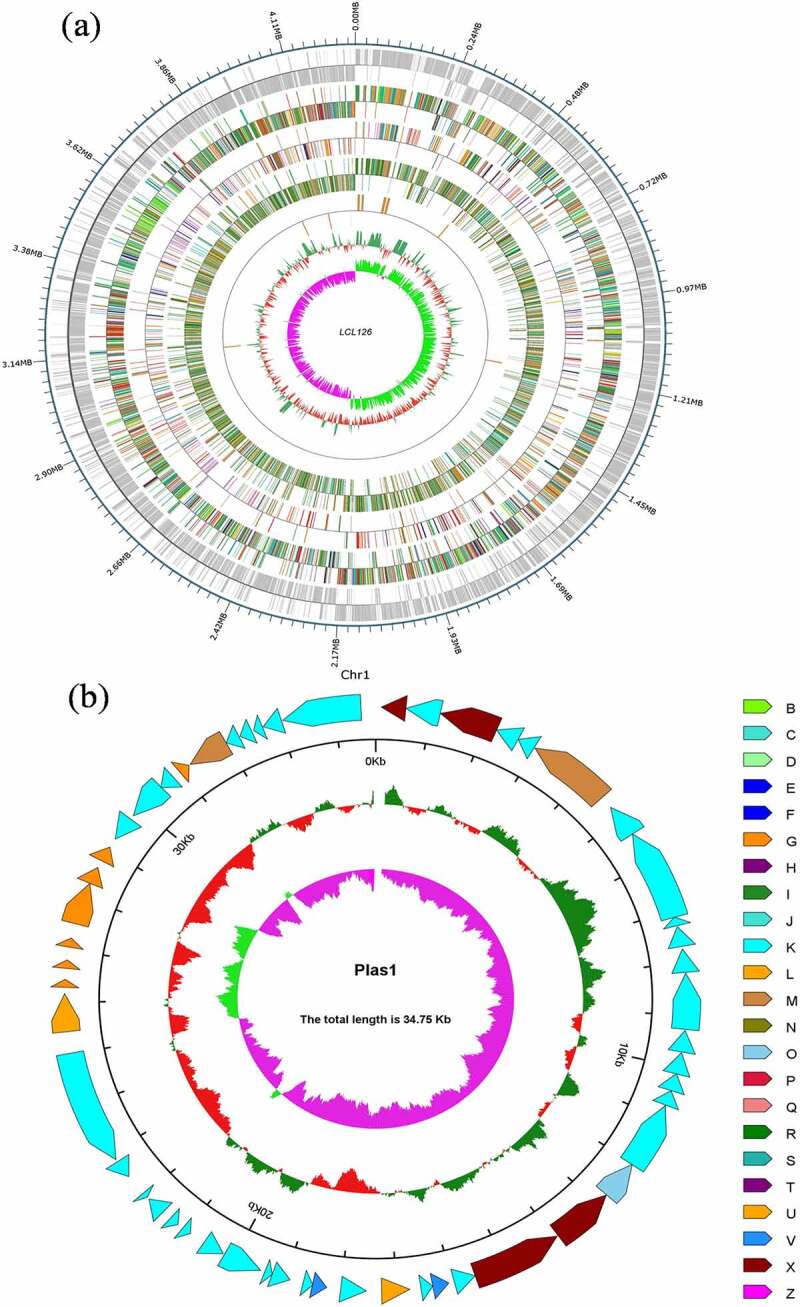
Whole-genome visualization map of *Clostridium difficile* LCL126. LCL126-Chr1 genome-wide map: the outermost circle is the position coordinates of the genome sequence, from the outside to the inside indicating the coding gene and the result of gene function annotation (a); LCL126-Plas1 genome-wide map: From the outside to the inside is the Cluster of Orthologous Groups (COG) functional annotation classification genes (the arrow indicates the positive chain code in a clockwise direction), the genome sequence position coordinates, and the genome GC content (b)

In biological systems, methylation is enzymatically catalyzed and involves heavy metal modification, gene expression regulation, protein function regulation, and ribonucleic acid (RNA) processing. The presence of methylation modification plays an important role in the substitution of DNA transcription, and abnormal methylation will cause many of diseases usually, thus the systematic mapping of methylation groups has attracted increasing interest. In the epigenetic modification, the final genome assembly results were detected by methylation sites and three types of modified sites were obtained, the number of modification sites type m4C, m5C, m6A, and modified base was 6989, 36,666, 3534, and 351,658, which account for 1.75%, 9.1930%, 0.8861%, and 88.1686%, respectively. The distribution statistics of methylation motifs on genetic regions (GRs)/intergenic region (IRGs) shows that the number of m4C, m5C, and m6C methylation types on the genome was 6989, 36,666, and 3534. The number of methylated types in a gene region account for 83.3, 16.69, and 78.04%. Additionally, the number of nucleotide motif sequence CAAAAA and TV recognized by unmethylated methyltransferase on the genome is 2927 and 1,782,530, and the number of unmethylated motif sequences on gene regions account 78.1 and 83.34%. Visualization of apparent modification distribution methylation circle diagram shown in Figure 3. Whole-genome visualization map is demonstrated in Figure 4 and the detailed information includes analysis of non-coding RNA, and gene function annotations were stated in the above first part of the genome overview component characteristics. Some strains were relevance to serious illness such as ribo-type 176, 244, 27, 17, 78 by encoding or destroying genes for different enzymes [14]. While as the separation of more detailed strains with the technology advances, it seems to be more diversification than previously record [34,35]. But no reports related to serious diseases found till now. Previous study described the number and type of the pro-inflammatory strain surface protein SlpA that may relate to toxic behavior in *C. difficile* [14,36,37]. The genomic information was provided in present study confirmed that *C. difficile* LCL126 has some relevant toxin genes, while in-depth study of its metabolome and functional genes needs to be further studied to explore the toxicological mechanism of *C. difficile* LCL126, aimed to improve the comprehensive understanding of microorganisms and promote the *C. difficile* related infectious diseases.

## 4. Conclusion

Genome sequencing could be revealing the importance and comprehensive information aspect of biology. Present study has profound insights into genomics and diversity of *C. difficile* bacteriophages, while this field was still at infancy. The *Clostridium difficile* LCL126 exist virulence-related gene including 22 increased virulence genes, it might be having virulence potential. In view of the increasing number of *C. difficile* cases and the severity of the disease, it was essential to expand current limited scientific knowledge about *C. difficile* bacteriophages. Finally, the ultimate purpose was to develop a non-antibiotic approach via the encode of antibacterial genes.

## Supplementary Material

Supplemental Material

## Highlights:

1. The whole genome information of *C. difficile* LCL126 was identified.

2. *C. difficile* LCL126 has total size of the genome 4,301,949 bp with a 27.97% of GC content.

3. 133 reduced virulence and 22 increased virulence (hypervirulence) was detected.

4. Methylation is expressed mainly distributed in m4C, m5C, and m6C with 6989, 36,666, and 3534 genes.

5. The strain of *C. difficile* LCL126 was predicted to have toxic potential.

## References

[1] Rineh A, Kelso MJ, Vatansever F, et al. Clostridium difficile infection: molecular pathogenesis and novel therapeutics. Expert Review of Anti-infective Therapy. 2014;12(1):131–150. 24410618 10.1586/14787210.2014.866515 PMC4306399

[2] Cao H, Wong S, Yam W, et al. Genomic investigation of a sequence type 67 Clostridium difficile causing community-acquired fulminant colitis in Hong Kong. Int J Med Microbiol. 2019;309(5):270–273. 31113737 10.1016/j.ijmm.2019.05.003

[3] Monot M, Eckert C, Lemire A, et al. Clostridium difficile: new insights into the evolution of the pathogenicity locus. Sci Rep. 2015;5(1):15023. 26446480 10.1038/srep15023 PMC4597214

[4] Marvaud JC, Quevedo-Torres S, Eckert C, et al. Virulence of new variant strains of Clostridium difficile producing only toxin A or binary toxin in the hamster model. Microbes Infect. 2019;32:100590. 10.1016/j.nmni.2019.100590 PMC6734109 31516714

[5] Alcala L, Reigadas E, Marín M, et al. Impact of clinical awareness and diagnostic tests on the underdiagnosis of Clostridium difficile infection. Eur J Clin Microbiol Infect Dis. 2015;34:1515–1525. 25904126 10.1007/s10096-015-2380-3

[6] Solomon SL, Oliver KB. Antibiotic resistance threats in the United States: stepping back from the brink. Am Fam Physician. 2014;89:938. 25162160

[7] Dupuy B, Matamouros S. Regulation of toxin and bacteriocin synthesis in Clostridium species by a new subgroup of RNA polymerase σ-factors. Res Microbiol. 2006;157:201–205. 16439101 10.1016/j.resmic.2005.11.004

[8] Knight DR, Elliott B, Chang BJ, et al. Diversity and evolution in the genome of Clostridium difficile. Clin Microbiol Rev. 2015;28:721–741. 26085550 10.1128/CMR.00127-14 PMC4475645

[9] Mani N, Dupuy B. Regulation of toxin synthesis in Clostridium difficile by an alternative RNA polymerase sigma factor. P Nat Acad Sci. 2001;98:5844–5849. 10.1073/pnas.101126598 PMC33301 11320220

[10] Wang S, Hong W, Dong S, et al. Genome engineering of Clostridium difficile using the CRISPR-Cas9 system. Clin Microbiol Infect. 2018;24:1095–1099. 29604353 10.1016/j.cmi.2018.03.026

[11] Bouillaut L, Dubois T, Sonenshein A, et al. Integration of metabolism and virulence in Clostridium difficile. Res Microbiol. 2015;166:375–383. 25445566 10.1016/j.resmic.2014.10.002 PMC4398617

[12] Sim JHC, Truong C, Minot SS, et al. Determining the cause of recurrent Clostridium difficile infection using whole genome sequencing. Diagn Micr Infec Dis. 2017;87(1):11–16. 10.1016/j.diagmicrobio.2016.09.023 27771207

[13] Zeng Z, Zhao H, Dorr MB, et al. Bezlotoxumab for prevention of Clostridium difficile infection recurrence: distinguishing relapse from reinfection with whole genome sequencing. Anaerobe. 2019;61:102137. 31846705 10.1016/j.anaerobe.2019.102137

[14] Quesada-Gómez C, Murillo T, Arce G, et al. Proteogenomic analysis of the Clostridium difficile exoproteome reveals a correlation between phylogenetic distribution and virulence potential. Anaerobe. 2020;62:102151. 31945474 10.1016/j.anaerobe.2020.102151

[15] Li Z, Liu X, Zhao J, et al. Comparison of a newly developed binary typing with ribotyping and multilocus sequence typing methods for Clostridium difficile. J Microbiol Methods. 2018;147:50–55. 29486225 10.1016/j.mimet.2018.02.012

[16] Riedel T, Wittmann J, Bunk B, et al. A Clostridioides difficile bacteriophage genome encodes functional binary toxin-associated genes. J Biotechnol. 2017;250:23–28. 28216103 10.1016/j.jbiotec.2017.02.017

[17] Lim HJ, Lee EH, Yoon Y, et al. Portable lysis apparatus for rapid single‐step DNA extraction of Bacillus. Portable lysis apparatus for rapid single‐step dna extraction of bacillus subtilis. J Appl Microbiol. 2016;120:379–387. 26606545 10.1111/jam.13011

[18] Reiner J, Pisani L, Qiao W, et al. Cytogenomic identification and long-read single molecule real-time (SMRT) sequencing of aBardet–Biedl Syndrome 9(BBS9) deletion. Npj Genom Med. 2018;3. 29367880 10.1038/s41525-017-0042-3 PMC5778042

[19] Besemer J, Lomsadze A, Borodovsky M. GeneMarkS: a self-training method for prediction of gene starts in microbial genomes. Nucleic Acids Res. 2001;29:2607–2618. 11410670 10.1093/nar/29.12.2607 PMC55746

[20] Hsiao W, Wan I, Jones SJ, et al. IslandPath: aiding detection of genomic islands in prokaryotes. Bioinformatics. 2003;19:418–420. 12584130 10.1093/bioinformatics/btg004

[21] Minoru M, Susumu G, Shuichi K, et al. The KEGG resource for deciphering the genome. Nucleic Acids Res. 2004;32:277–280. 10.1093/nar/gkh063 PMC308797 14681412

[22] Minoru M, Susumu G, Masahiro H, et al. From genomics to chemical genomics: new developments in KEGG. Nucleic Acids Res. 2006;34:354–357. 10.1093/nar/gkj102 PMC1347464 16381885

[23] Galperin MY, Makarova KS, Wolf YI, et al. Expanded microbial genome coverage and improved protein family annotation in the COG database. Nucleic Acids Res. 2015;43:261–269. 10.1093/nar/gku1223 PMC4383993 25428365

[24] Cantarel BL, Coutinho PM, Corinne R, et al. EnZymes database (CAZy): an expert resource for glycogenomics. Nucleic Acids Res. 2009;37:233–238. 10.1093/nar/gkn663 PMC2686590 18838391

[25] Petersen TN, Brunak S, Heijne G, et al. SignalP 4.0: discriminating signal peptides from transmembrane regions. Nat Methods. 2011;8:785–786. 21959131 10.1038/nmeth.1701

[26] Valerie E, Thomas N, Alexander P, et al. DB—updates and novel features for a better annotation of bacterial secreted proteins and type III, IV, VI secretion systems. Nucleic Acids Res. 2016;44:669–674. 26590402 10.1093/nar/gkv1269 PMC4702896

[27] Martin U, Rashmi P, Arathi R, et al. Interactions database (PHI-base): additions and future developments. Nucleic Acids Res. 2015;43:645–655. 25414340 10.1093/nar/gku1165 PMC4383963

[28] Krzywinski M, Schein J, Birol I, Connors J, Gascoyne R, Horsman D, Jones S, Marra M. Circos: an information aesthetic for comparative genomics. Genome Res. 2009; 1:1639–1645. 10.1101/gr.092759.109 PMC2752132 19541911

[29] Holt K, Seth-Smith H, Quail M, et al. Evolutionary dynamics of Clostridium difficile over short and long time scales. Proc Natl Acad Sci. 2010;107:7527–7532. 20368420 10.1073/pnas.0914322107 PMC2867753

[30] Forgetta V, Oughton MT, Marquis P, et al. Fourteen-genome comparison identifies DNA markers for severedisease- associated strains of Clostridium difficile. J Clin Microbiol. 2011;49:2230–2238. 21508155 10.1128/JCM.00391-11 PMC3122728

[31] Xu P, Zhang X, Su H, et al. Genome-wide analysis of PYL-PP2C-SnRK2s family in Camellia sinensis. Bioengineered. 2020;11:103–115. 31903833 10.1080/21655979.2019.1710932 PMC6961588

[32] Kuehne S, Minton N. ClosTron-mediated engineering of Clostridium. Bioengineered. 2012;3:247–254. 22750794 10.4161/bioe.21004 PMC3476875

[33] Andres-Lasheras S, Bolea R, Mainar-Jaime R, et al. Presence of Clostridium difficile in pig faecal samples and wild animal species associated with pig farms. J Appl Microbiol. 2017;122:462–472. 27990723 10.1111/jam.13343

[34] Kang Z, Zhang J, Jin P, et al. Directed evolution combined with synthetic biology strategies expedite semi-rational engineering of genes and genomes. Bioengineered. 2015;6:136–140. 25621864 10.1080/21655979.2015.1011029 PMC4601291

[35] Sheehan D, O’Sullivan S. Online homology modelling as a means of bridging the sequence-structure gap. Bioengineered. 2011;2:299–305. 10.4161/bbug.2.6.16116 22064508

[36] Janoir C. Virulence factors of Clostridium difficile and their role during infection. Anaerobe. 2016;37:13–24. 26596863 10.1016/j.anaerobe.2015.10.009

[37] Tan Y, Yang X, Pei M, et al. A genome-wide survey of interaction between rice and Magnaporthe oryzae via microarray analysis. Bioengineered. 2021;12:108–116. 33356807 10.1080/21655979.2020.1860479 PMC8806351

