# Supplementary material for: Complete genome sequence of the Clostridium difficile LCL126
Source: Bioengineered. 2021 Apr 25;12(1):745–54. doi: 10.1080/21655979.2021.1894798 (PMC8806205; doi:10.1080/21655979.2021.1894798)
Supplement: Supplemental Material [file KBIE_A_1894798_SM4969.docx]

**SUPPLEMENTARY INFORMATION**

**Figure captions:**

**Fig.S1:** Gene function analysis of *Clostridium difficile* LCL126 based on Cluster of Orthologous Groups (COG) of proteins (a), Carbohydrate-Active enZYmes (b), and Pathogen Host Interactions Database (c), the abscissa indicates the type of classed function, and the ordinate indicates the number of genes on the annotation.

**Fig. S1.**
